# Creatine Prevents the Structural and Functional Damage to Mitochondria in Myogenic, Oxidatively Stressed C2C12 Cells and Restores Their Differentiation Capacity

**DOI:** 10.1155/2016/5152029

**Published:** 2016-08-17

**Authors:** Elena Barbieri, Michele Guescini, Cinzia Calcabrini, Luciana Vallorani, Anna Rita Diaz, Carmela Fimognari, Barbara Canonico, Francesca Luchetti, Stefano Papa, Michela Battistelli, Elisabetta Falcieri, Vanina Romanello, Marco Sandri, Vilberto Stocchi, Caterina Ciacci, Piero Sestili

**Affiliations:** ^1^Department of Biomolecular Sciences, University of Urbino Carlo Bo, 61029 Urbino, Italy; ^2^Interuniversity Institute of Myology (IIM), Urbino, Italy; ^3^Department for Life Quality Studies, Alma Mater Studiorum, University of Bologna, 47921 Rimini, Italy; ^4^Department of Biomedical Sciences, University of Padova, Venetian Institute of Molecular Medicine, 35129 Padova, Italy

## Abstract

Creatine (Cr) is a nutritional supplement promoting a number of health benefits. Indeed Cr has been shown to be beneficial in disease-induced muscle atrophy, improve rehabilitation, and afford mild antioxidant activity. The beneficial effects are likely to derive from pleiotropic interactions. In accord with this notion, we previously demonstrated that multiple pleiotropic effects, including preservation of mitochondrial damage, account for the capacity of Cr to prevent the differentiation arrest caused by oxidative stress in C2C12 myoblasts. Given the importance of mitochondria in supporting the myogenic process, here we further explored the protective effects of Cr on the structure, function, and networking of these organelles in C2C12 cells differentiating under oxidative stressing conditions; the effects on the energy sensor AMPK, on* PGC-1α*, which is involved in mitochondrial biogenesis and its downstream effector* Tfam* were also investigated. Our results indicate that damage to mitochondria is crucial in the differentiation imbalance caused by oxidative stress and that the Cr-prevention of these injuries is invariably associated with the recovery of the normal myogenic capacity. We also found that Cr activates AMPK and induces an upregulation of* PGC-1α* expression, two events which are likely to contribute to the protection of mitochondrial quality and function.

## 1. Introduction

Over the last few decades, the interest in creatine (Cr) supplementation has increased notably. Cr has been used in different conditions, not only in improving skeletal muscle energy homeostasis [1–3] but also as a potential therapeutic agent for subjects suffering from muscle wasting and myopathies [4–8].

Cr supplementation has been shown to increase satellite cells and myonuclei number in human skeletal muscle, especially if associated with contractile activity [9–11]. The mechanisms by which Cr affects the growth and differentiation of myogenic cells are complex and probably not yet completely understood [12]. In 1972, Ingwall et al. [13] reported that Cr increases the expression of myosin heavy chain and induces muscle-specific protein synthesis in both skeletal and cardiac chicken myotubes in culture. Previous works by our group and others on C2C12 myogenic cells have shown that Cr stimulates growth and protein accretion, associated with an increase in insulin-like growth factor 1 (*IGF-1*) mRNA and coordinated upregulation of myogenic regulatory factors mRNA [14, 15]. In parallel with these positive effects in normal conditions, we also reported that pretreatment with Cr, through multiple converging actions, prevents the inhibition of C2C12 myoblasts' differentiation into mature myotubes caused by oxidative stress [15]. The oxidative-dependent actions on C2C12 differentiating cells leading to a decrease of their cellular viability and to the myogenic arrest are complex [15, 16]. Among other toxicological relevant events, we noticed severe ultrastructural damage to mitochondria suggesting that they could represent critical targets of oxidative attack and, interestingly, supplemental Cr was capable of preserving the integrity of mitochondrial ultrastructure [15]. In this regard, it is worth noting that Cr has been shown to protect mitochondria in other cell types exposed to various stressing conditions [17–19].

A growing body of evidence shows that mitochondria act as potential regulators of myogenesis and several studies suggest that impairment of mitochondrial structure and activity blocks myogenic differentiation. Indeed, mitochondrial-targeting chemicals, such as antimycin, azide, chloramphenicol, carbonyl cyanide, m-chlorophenylhydrazone carbonyl cyanide, p-(trifluoromethoxy)phenylhydrazone, ethidium bromide, myxothiazol, rotenone, and oligomycin tetracycline [20–30], inhibit the myogenic process. Moreover, it has been observed that respiration-deficient myoblasts lacking mitochondrial DNA—rho° cells—fail to differentiate into myotubes [22, 23]. Thus, the preservation of the mitochondrial integrity and functionality in differentiating myoblasts is necessary to complete the myogenic process in myotube maturation.

In the present study, we investigated the effect of Cr on oxidative-induced damage with a particular attention to mitochondrial ultrastructure, subcellular localization, and function in the early- (24 h after serum removal, after exposure to H_2_O_2_) and middifferentiation phase (myotubes containing more than two nuclei, 48 or 72 h after serum removal) of myogenesis in C2C12 cells.

Our findings provide insights into the mechanisms underlying the Cr protection of mitochondrial damage inflicted by H_2_O_2_ intoxication of C2C12 cells and into the importance of this beneficial effect in preserving their myogenic capacity.

## 2. Materials and Methods

### 2.1. Cell Culture and Treatment Conditions

#### 2.1.1. Cell Culture

C2C12 mouse myoblasts (Sigma-Aldrich, Italy) were cultured in DMEM (Sigma-Aldrich, Italy) supplemented with 10% v/v fetal bovine serum (Sigma-Aldrich, Italy), 2 mM glutamine (Sigma-Aldrich, Italy), and antibiotics (50 U/mL penicillin, 50 *μ*g/mL streptomycin, Sigma-Aldrich, Italy) for 24–48 h to reach 80% of cell confluence. C2C12 cell differentiation was induced by serum withdrawal 1% v/v (Differentiating Medium, DM). Myogenic differentiation was performed as described in [15]. Cells were observed and processed for the experiments at critical time intervals, that is, at selected differentiation days (DifD), which are referred to in the text as “DifD*n*,” where “*n*” is the elapsed time, in days, from the commencement of differentiation.

#### 2.1.2. Treatment Conditions

Cr preloading of C2C12 was carried out by adding 3 mM or 10 mM Cr (Creapure, AlzChem, Germany) to myoblasts over the first 24 h of differentiation. At this time point (i.e., at the end of DifD1), Cr-supplemented or Cr-unsupplemented cultures were exposed to 0 or 0.3 mM H_2_O_2_ for 1 h in serum-free medium and then cultured in Cr- and oxidant-free, DM up to day 3 (DifD3), when fusing cells were forming the first myotubes with more than two nuclei. Trolox (Sigma-Aldrich, Italy, 0.1 mM) was used as a positive control for the antioxidant effect as described in Sestili et al. [15]. Aminoimidazole-4-carboxamide ribonucleotide (AICAR, Sigma-Aldrich, Italy) was used (2 mM for 1 h) as a positive control for AMPK activation [31].

#### 2.1.3. Estimation of Myogenic Index

The myogenic index was determined as reported elsewhere [32] and evaluated as the number of nuclei in myotubes divided by the total number of nuclei in myoblasts and myotubes. Twenty optical fields were randomly chosen.

#### 2.1.4. Determination of Cell Viability

Cytotoxicity was determined using the trypan blue exclusion assay as described in Sestili et al. [15]. Results were expressed as per cent survival.

### 2.2. Analysis of Mitochondrial Properties

Mitochondria were analysed using* in situ* (permeabilized fixed and live cells) and* in vitro* (isolated mitochondria) approaches to study mitochondrial morphology, distribution, dynamics, and function under defined substrate conditions.

#### 2.2.1. Mitochondrial Ultrastructure

For transmission electron microscopy (TEM) analysis, C2C12 monolayers were fixed with 2.5% glutaraldehyde in PBS for 5 min, gently scraped, and centrifuged. Pellets were fixed again in a new fixative solution for 30 min, OsO_4_ postfixed, alcohol-dehydrated, and araldite embedded as reported in [33]. Thin sections were collected on nickel 300-mesh grids and stained with uranyl acetate and lead citrate. The observations were carried out with a Philips CM10 transmission electron microscope (FEI Company) at 80 KV.

#### 2.2.2. Live Cell Imaging Using Confocal Laser Scanning Microscopy

For mitochondria and lysosome confocal live imaging, cells were grown on MatTek glass bottom chambers (MatTek Corporation, Ashland, MA) costained with 100 nM MitoTracker red (MTR, Molecular Probes, Italy) and 500 nM LysoTracker green (LTG, Molecular Probes, Italy) and incubated for 30 min according to Luchetti et al. [34]. The incubation was performed at 37°C in 5% CO_2_. Labelled mitochondria were observed by confocal microscopy on a Leica TCS SP5 II (Leica Microsystems, Italy) instrument and ImageJ software (NIH, Bethesda, MD).

Each sample was examined through successive optical slices (0.2 mm each) along the *z*-axis, and the images obtained were postprocessed applying Wiener filter deconvolution. After deconvolution, the images were further processed in ImageJ software (NIH, Bethesda, MD) using the JACoP plug-in for colocalization studies. We used Pearson's coefficient as the parameter to measure colocalization in our samples.

#### 2.2.3. Mitochondrial Membrane Potential and Mass

Mitochondrial membrane potential (ΔΨ) was quantified cytofluorimetrically according to Salucci et al. [35] by measuring the extent of uptake of the potentiometric dye, tetramethyl rhodamine methyl ester (TMRE, Sigma-Aldrich, Italy), normalized to MitoTracker Green (MTG), which is taken up by mitochondria independently of membrane potential. During the acute exposure to H_2_O_2_ at DifD1, myogenic C2C12 cells were stained with 100 nM MTG for 30 min and in the last 15 min were coloaded with 50 nM TMRE at 37°C. Cells were observed immediately after the oxidative injury. To verify eventual hyperpolarization in case of mitochondrial dysfunction, cells were tested for their sensitivity versus oligomycin, a specific inhibitor of F0-part of H^+^ ATP synthase, and samples were analysed with and without 5 *μ*M oligomycin (Sigma-Aldrich, Italy) for 1 h, as described in Romanello et al. [36]. The protonophore carbonyl cyanide p-trifluoromethoxyphenylhydrazone (FCC, 4 mM) was then added to the cell culture medium. Fluorescent intensity in MTG was measured to estimate the overall mitochondrial mass.

#### 2.2.4. Analysis of Cardiolipin Content/Peroxidation

C2C12 cells were directly labelled in a well with 100 nM 10-N-nonyl acridine orange (NAO), for 30 min at 37°C in 5% CO_2_. NAO reflects total mitochondrial mass independently of activity because it binds to cardiolipin in the mitochondrial membrane. Following incubation, cells were harvested and 300 *μ*L of fresh medium was added. Changes in mitochondrial fluorescence intensity were analysed by FACScan flow cytometer equipped with CellQuest software (BD Biosciences, Italy) as described in [37].

#### 2.2.5. Immunoblotting

Cells were lysed and immunoblotted as described earlier [15]. Blots were stripped using Restore Western Blotting Stripping Buffer (Amersham Pharmacia, Italy) and reprobed if necessary. The following primary antibodies from Cell Signaling were used: anti-AMPK, anti-phospho-AMPK (Thr 172), anti-phospho-ACC (Ser 79), and anti-*α*-tubulin (Sigma-Aldrich, Italy).

#### 2.2.6. Mitochondrial Biogenesis

Cells cultured and treated as described above were analysed for the expression of specific mRNAs at DifD0, DifD1, DifD2, and DifD3. At each differentiation time, plates were washed with PBS; total DNA and RNA were extracted from cell cultures using QIAamp DNA and RNeasy Mini Kit (Qiagen, Germany), respectively, according to the manufacturer's instructions. All quantitative real-time PCR reactions were carried out in the StepOne Plus (Applied Biosystems, Italy) using 2x SYBR Select Master Mix (Life Technologies, Italy) and the PCR conditions were already described in Sestili et al. [15] and Barbieri et al. [33]. The relative gene expressions of peroxisome proliferator-activated receptor gamma coactivator 1-alpha (*PGC-1α*) and mitochondrial transcription factor A (*Tfam*) were quantified using 1 *μ*L of cDNA template; the amount of each target transcript was related to that of the gene encoding ribosomal protein S16. The mtDNA/nDNA ratio was obtained as reported in Barbieri et al. [33], relating the mitochondrial and nuclear DNA quantities amplifying both* COXII* and* GAPDH* as mtDNA and nDNA targets, respectively. All oligonucleotide primers have been previously described in Barbieri et al. [33].

#### 2.2.7. Mitochondrial Proteomic Analysis

Mitochondria from 3 × 10^7^ cells were extracted as previously described [33] and suspended in 8 M urea, 4% CHAPS, 65 mM DTE, and 40 mM Tris base and sonicated for 5 s on ice. Following centrifugation at 21000 g, protein concentration was determined by Bradford assay [38] and 80 *μ*g of total proteins used for 2-dimensional electrophoresis (2DE). Analytical gels were stained with silver nitrate [39], while semipreparative gels for mass spectrometry analysis were stained with Brilliant Blue R250 (Sigma-Aldrich, USA). Gel images were acquired by Fluor-S MAX multi-imaging system (Bio-Rad Laboratories Italy, Segrate, Italy), and the data were analysed with ImageMaster 2D Platinum software. Gel digestion was carried out according to Shevchenko's protocol [40] and LC-ESI-MS/MS analysis was performed using a Q-TOF micro*™* mass spectrometer (Micromass, UK) as previously described [41].

#### 2.2.8. Statistical Analysis

Unless noted otherwise, the results were expressed as mean values ± SD. The significance of differences between the mean values recorded for different experimental conditions was calculated by Student's *t*-test and *P* values are indicated where appropriate in the figures and their legends.

With respect to the determination of ΔΨ, cardiolipin, AMPK, ACC,* PGC-1a*, and* Tfam* levels and mtDNA copy numbers, the effects of all treatments were tested using a two-tailed one-way ANOVA analysis. Tukey's* post hoc* test was performed using GraphPad Prism version 5.00 for Windows (GraphPad Software, USA). The significance threshold was set to 0.05.

## 3. Results

### 3.1. Effects of Cr on Mitochondrial Ultrastructure of H_2_O_2_-Treated C2C12 Cells

The effect of oxidant injury (0.3 mM H_2_O_2_ for 1 h) on the viability and myogenic index of C2C12 myoblasts with or without Cr supplementation (3 or 10 mM for 24 h) committed to differentiate was first determined. Oxidative challenge resulted in a reduction of cell survival (67.3% ± 5.3 24 h after intoxication) and in severe inhibition of myogenic differentiation of surviving cells (with a myogenic index of 0.081 ± 0.0072 as compared to 0.51 ± 0.047 of controls): both events could be prevented by 3 mM Cr supplementation (survival of 93.3% ± 8.4 and a myogenic index of 0.45 ± 0.039). 10 mM Cr yielded results not significantly different as compared to 3 mM; therefore, the lower concentration was used in subsequent experiments.

**Figure 1:**
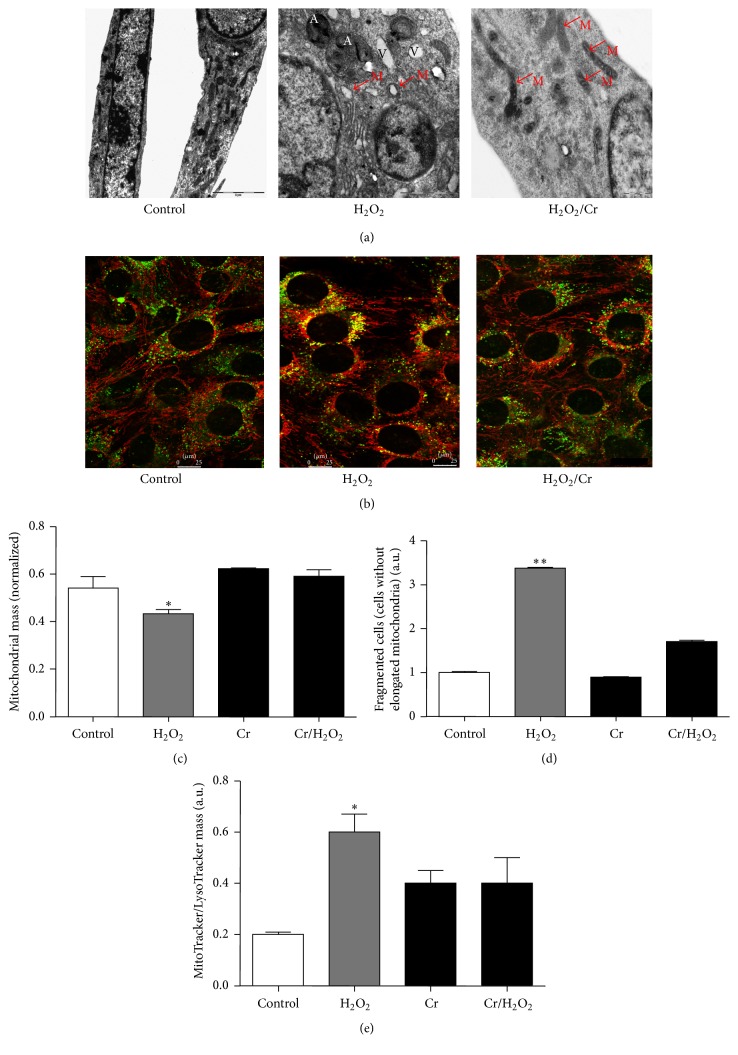
Protective effect of Cr on oxidant-injured mitochondria in differentiating C2C12 myoblasts. TEM micrographs and confocal images were taken immediately after oxidative stress at DifD1. Images were representative of five independent experiments. (a) H_2_O_2_ treatment induced cytoplasmic vacuolization, mitochondrial swelling, and disruption; Cr prevented the effect of H_2_O_2_: Cr-supplemented cells appeared comparable to the controls, showing lower cytoplasmic vacuolization and numerous elongated mitochondria with perfectly preserved cristae. Bar = 2, 1, and 0.5 *μ*m specified in each micrograph. V: vacuole; A: autophagosome; M: mitochondrion. (b) Confocal microscopy of C2C12 myoblasts after double staining with MitoTracker (MTR) and LysoTracker (LTG) showing the mitochondrial network morphology and lysosome distribution within myocytes and the effect of oxidative challenge with or without Cr supplementation. Bar = 25 *μ*m. (c) Analysis of confocal images showing the quantification of mitochondrial mass. (d) Number of cells with fragmented mitochondria. (e) Extent of colocalization of MTR and LTG. Data are the means ± SD. ^*∗*^ *P* ≤ 0.05; ^*∗∗*^ *P* ≤ 0.01 compared to control (*n* = 5).

The effects of supplemental Cr on the mitochondrial ultrastructural damage in this intoxication setting were then studied with TEM.  Figure 1(a) shows TEM micrographs taken at DifD1, immediately after oxidative insult; H_2_O_2_ treatment induced severe ultrastructural damage to the majority of the mitochondria, which showed typical matrix swelling and loss of cristae. Notably, Cr supplementation protected mitochondria from these injuries: their number was comparable to that of controls and they displayed an elongated shape and well-preserved cristae. We also observed distinctive H_2_O_2_-mediated cytoplasmic vacuolization, typical of myocytes' response to oxidative insult [15]. We likewise observed several autophagic vacuoles with double membranes containing disorganised mitochondrial cristae; sometimes, autophagic vacuoles occurred close to the sarcolemma. Again, Cr treatment prevented the formation of these cytoplasmic vacuoles. 

Going back to viability and myogenic index, according to previous data [15], addition of Trolox (an established radical scavenger included as a positive control) resulted in a qualitatively and quantitatively different protective effect as compared to Cr against H_2_O_2_ challenge, in that it prevented C2C12 cells' decrease in viability (cell survival of 91.2% ± 6.4 versus 67.3% ± 5.3 of H_2_O_2_-only-treated cells) but was unable to restore their differentiation capacity (a myogenic index of 0.20 ± 0.019 as compared to 0.45 ± 0.039 of H_2_O_2_-injured, Cr-supplemented cells and 0.51 ± 0.047 of controls).

### 3.2. Effect of Cr on Mitochondrial Morphology

Confocal images in Figure 1(b) show filamentous mitochondria with well-defined tubular shapes in control cells, while H_2_O_2_ treatment caused an increase in fragmented, round, and perinuclear-located mitochondria. Interestingly, Cr preloading prevented these morphological alterations; indeed, mitochondria maintained their filamentous and tubular shape paralleling the myocytes' elongation axis typical of controls. Cells preloaded with Cr* per se* present a mitochondrial profile identical to controls (not shown).

The analysis of the fluorescence intensity of high definition confocal images allowed us to quantitate mitochondrial mass at the early differentiation phase (immediately after H_2_O_2_ insult, DifD1, Figure 1(c)); results demonstrated a significant decrease of mitochondrial mass in oxidative damaged myocytes. By contrast, Cr preloading of H_2_O_2_-treated cells prevented this effect.

Further analysis (see Section 2.2.2) of the above images showed that, in comparison, H_2_O_2_ treatment led to a significant increase in the number of fragmented mitochondria with small perinuclear ring shapes. The cells showing 70% fragmented or intermediate mitochondria were classified as fragmented cells [42]; their number increased significantly upon infliction of oxidative treatment and, not surprisingly, Cr preloading prevented this effect (Figure 1(d)).

In these oxidative stressing conditions, lysosomes are likely to be actively involved in the removal of damaged mitochondria, as suggested by the appearance of autophagosomes revealed by TEM (Figure 1(a)). To strengthen this point, the degree of mitochondria sequestration by lysosomes was quantified by performing a double staining protocol with MTR and LTG, followed by quantification of the lysosomal and mitochondrial overlapping. This analysis showed that oxidative stress caused intense perinuclear colocalization of lysosomes with mitochondria (Figure 1(e)), a finding in line with the ultrastructural TEM observations. Not surprisingly, Cr was found to consistently prevent the lysosomal/mitochondrial colocalization and to preserve the diffuse localization of lysosomes within the cytoplasm. Notably, the established* bona fide* antioxidant Trolox showed a much weaker protective effect than Cr (not shown). Remarkably, in Cr-only-supplemented cells, well-preserved, mostly elongated mitochondria could be observed (not shown).

### 3.3. Effect of Cr on Mitochondrial Membrane Potential

**Figure 2:**
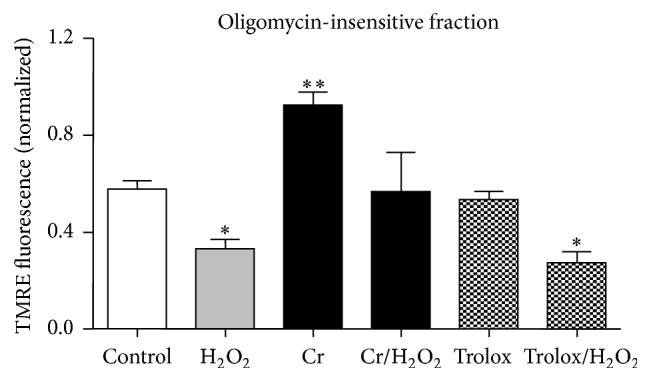
Protective effect of Cr on mitochondrial ΔΨ after oxidative challenge in differentiating C2C12 myoblasts. The mitochondrial ΔΨ (normalized ratio TMRE/MTG) was evaluated immediately after oxidative stress (DifD1) as the mitochondrial fraction insensitive to oligomycin. Data are the means ± SD. ^*∗*^ *P* ≤ 0.05; ^*∗∗*^ *P* ≤ 0.01 compared to control (*n* = 5).

Mitochondrial membrane potential per mitochondrial unit was studied cytofluorimetrically at DifD1 determining their TMRE/MTG ratio, which reflects the extent of ΔΨ, in the presence of oligomycin (5 *μ*M; see Section 2.2.3) [36]. In particular, our analysis (Figure 2) indicates a significant reduction of TMRE/MTG ratio (oligomycin-insensitive fraction) in oxidatively injured cells as compared to controls, an effect that could be prevented by Cr supplementation; Cr-only-treated myoblasts showed a significant increase in the TMRE/MTG ratio. Notably, Trolox, unlike Cr, failed to prevent the loss of ΔΨ in oxidatively injured cells.

### 3.4. Effect of Cr on Cardiolipin Peroxidation

**Figure 3:**
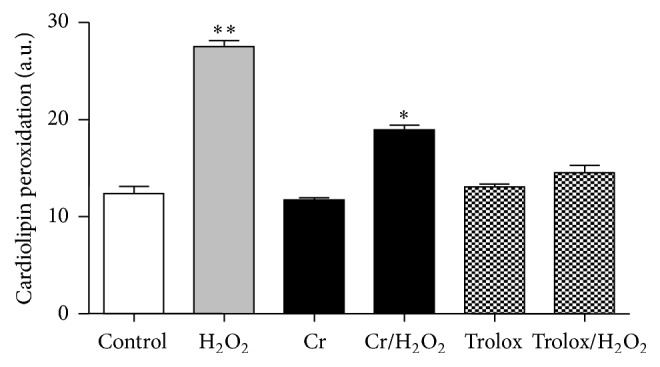
Protective effect of Cr on cardiolipin peroxidation induced by oxidative challenge in differentiating C2C12 myoblasts. Flow cytometric analysis of the cardiolipin-sensitive probe 10-nonyl acridine orange (NAO) was used to monitor changes in mitochondrial lipid peroxidation in differentiating oxidatively challenged C2C12 cells pretreated with 0 or 3 mM Cr. Analysis was performed at DifD2. Data are the means ± SD. ^*∗*^ *P* ≤ 0.05; ^*∗∗*^ *P* ≤ 0.01 compared to control (*n* = 5).

Flow cytometric analysis of NAO/cardiolipin interaction (a reliable marker of mitochondrial oxidative damage) showed that H_2_O_2_ treatment caused significant peroxidation of cardiolipin within 24 h after H_2_O_2_ challenge (Figure 3), an effect which could be significantly hampered by Cr, which afforded a 32% protection rate; Trolox was slightly more effective in preventing cardiolipin peroxidation.

### 3.5. Effect of Cr on the Expression of AMPK

**Figure 4:**
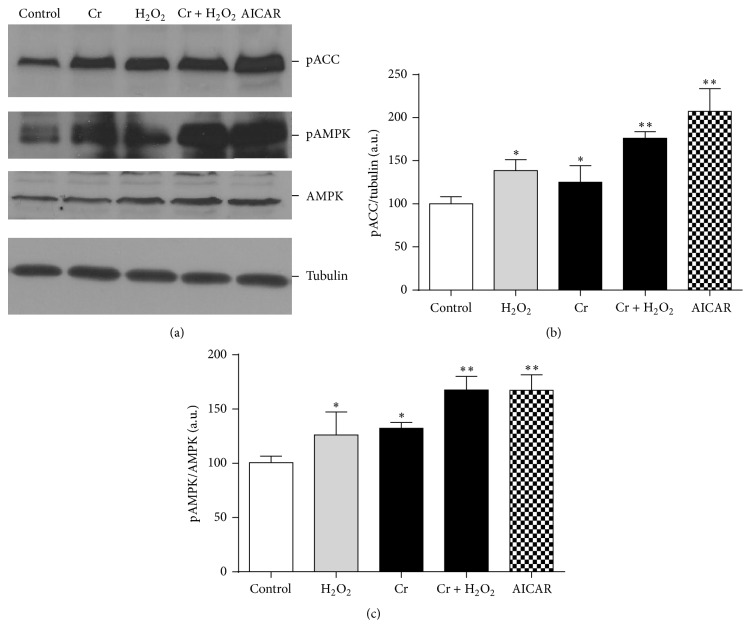
Effect of Cr on AMPK. (a) The cell lysates were separated by SDS-PAGE and analysed for pACC, pAMPK, and AMPK protein expression by Western blotting after the oxidative insult (DifD1); representative images were reported. Blots were also probed for tubulin protein as loading control. (b) The levels of phosphorylated AMPK versus total AMPK and (c) the levels of phosphorylated ACC versus tubulin were examined. Data are the mean ± SEM. ^*∗*^ *P* ≤ 0.05; ^*∗∗*^ *P* ≤ 0.01 compared to control (*n* = 4).

Figure 4(a) shows that Cr preloading increased the extent of phosphorylation of AMPK and of its downstream firmly established substrate acetyl-CoA carboxylase (ACC). H_2_O_2_ treatment,* per se*, also induced AMPK activation, in accordance with literature data [43]. Interestingly, in Cr-supplemented, oxidatively treated cells, the increase of AMPK phosphorylation was higher as compared to Cr-only- or H_2_O_2_-only-treated conditions, suggesting that the two agents act in a somehow additive fashion. A similar behaviour could be observed with regard to ACC phosphorylation (Figure 4). It is of note that AICAR, a reference AMPK activator, induced a robust increase of its phosphorylation in our cellular setting.

### 3.6. Effect of Cr on Mitochondrial Biogenesis and mtDNA Content

**Figure 5:**
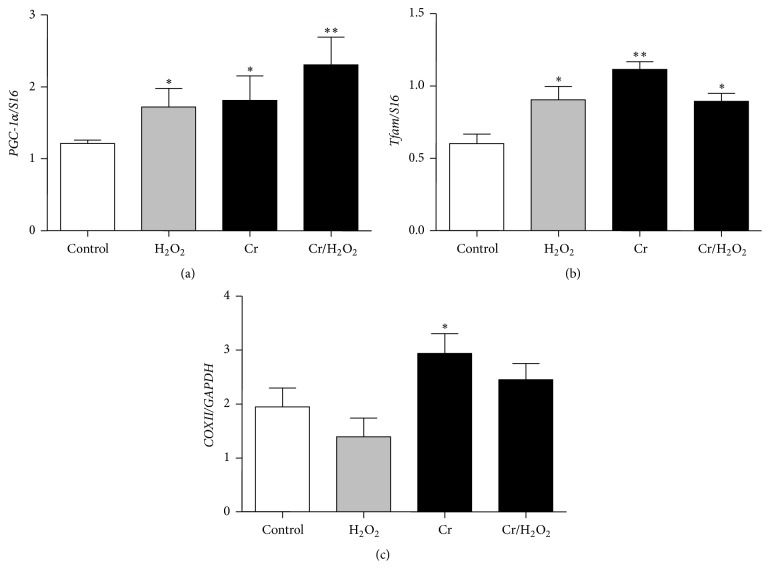
Effect of Cr on mitochondrial biogenesis under normal or oxidatively stressing conditions. mRNA expression level of* PGC*-*1α* (a) and of* Tfam* (b) at DifD1; mtDNA content at DifD3 (c). Quantitative analysis of* PGC-1α* and* Tfam* was performed by real-time PCR, and the amount of each target transcript was related to that of the reference gene (the ribosomal protein* S16*). The mtDNA content was determined by real-time PCR and expressed as mtDNA/nDNA ratio (*COXII/GAPDH*). Data are expressed as the mean ± SEM. ^*∗*^ *P* ≤ 0.05; ^*∗∗*^ *P* ≤ 0.01 compared to control (*n* = 5).

*PGC-1α* represents a master regulator of mitochondrial biogenesis by interacting with several transcription factors also crucial for maintaining mitochondrial DNA integrity [44, 45]. Figures 5(a) and 5(b) show the transcription level of both* PGC-1α* and* Tfam,* quantified using RT real-time PCR at DifD1. As shown in Figure 5(a), analysis of C2C12 cells treated with H_2_O_2_ or H_2_O_2_ + Cr showed that their* PGC-1α* expression levels were markedly increased as compared to controls. Similar to* PGC-1α*, the* Tfam* expression level during myoblasts differentiation was characterized by a tendency to increase (Figure 5(b)) as compared to controls. MtDNA copy number can be considered as another marker of mitochondrial biogenesis and, in general, of muscle health [46]. Our results show that, at DifD3, the relative mtDNA copy number in Cr-loaded and H_2_O_2_-injured cells was significantly higher as compared to Cr-free C2C12 cells exposed to H_2_O_2_ (Figure 5(c)).

### 3.7. Mitochondrial Proteomic Analysis

**Figure 6:**
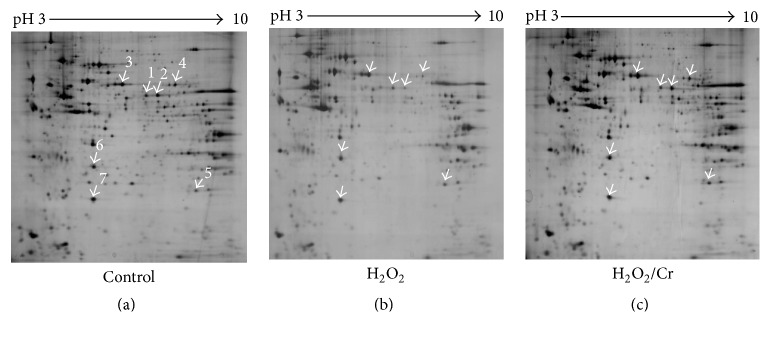
Effect of Cr on the mitochondrial proteomic pattern. Mitochondrial proteomic mapping from myogenic C2C12 cells at DifD3; (a) control cells; (b) H_2_O_2_-treated cells; and (c) Cr-supplemented myocytes treated with H_2_O_2_ as in (b). The arrows show differentially expressed proteins after H_2_O_2_ treatment. The major differences in H_2_O_2_-treated cells as compared to (a) and (c) were found in 1 and 2, mitochondrial aldehyde dehydrogenase-2; 3, protein disulfide isomerase; 4, dihydrolipoamide dehydrogenase; 5, Mn superoxide dismutase; 6, prohibitin; and 7, ATP synthase D chain. Images were representative of five independent experiments.

We next studied the mitochondrial proteome in C2C12 differentiating myoblasts using a 2D approach described previously [33]. We found that the 2D profile of H_2_O_2_-injured mitochondria (Figure 6(b)) was significantly different as compared to that of controls (Figure 6(a)); in particular, a significant reduction—or even the disappearance—of several proteins could be observed, especially in the pH range 4–7. By contrast, Cr supplementation afforded a nearly complete protection from these alterations and produced a protein pattern almost identical to control (Figure 6(c)), including the proteins whose expression significantly increases over differentiation [33]. In particular, the increased expression of dihydrolipoamide dehydrogenase (spot 4) and ATP synthase D chain (spot 7), both involved in energy production, reflects the capacity of preserving mitochondrial function.

## 4. Discussion

In a previous study, we showed that Cr protects differentiating C2C12 myoblasts from H_2_O_2_-dependent myogenic arrest [15] and that in oxidative stressing conditions, Cr, through multiple and converging mechanisms (increased expression of myogenic factors and of IGF-1, reduction of GSH consumption and of cell death through its antioxidant activity, and amelioration and preservation of cellular energy charge through increased availability of Cr Phosphate, CrP), might provide myoblasts with an enhanced adaptive potential resulting in the preservation of myogenic capacity.

Here, we demonstrate that Cr effectively and invariably protects mitochondrial structure and function of differentiating myoblasts from the damage inflicted by exogenously added H_2_O_2_ (0.3 mM for 1 h, DifD1), providing novel insights into the cellular mechanisms of Cr in preventing the myogenic arrest caused by oxidative challenge.

Among the cellular targets of oxidative attack, ultrastructural TEM analysis showed and confirmed that mitochondria appear to be seriously damaged and, interestingly, Cr was capable of preserving their integrity. Mitochondria have been recognized as crucial players in myogenesis, and a considerable body of evidence suggests that impairment of their subcellular organisation and activity blocks myogenic differentiation [15, 26, 30, 47, 48]. Our data further confirm this notion and also highlight the importance of preserving mitochondrial integrity in this differentiation process.

We adopted a morphofunctional and biochemical approach to study the mitochondrial structural organisation, specific mitochondria-targeted signaling induced by Cr, and other related functions using the C2C12 myogenic cellular model.

We found that oxidative insult induced multiple damage to muscle mitochondria, such as ultrastructural loss of cristae, osmotic swelling, networking destruction, decrease in mitochondrial mass, mitochondrial impaired function (ΔΨ), reduction of mtDNA copy number, increase in cardiolipinperoxidation, and quantitative modification of mitochondrial protein expression pattern. With regard to the fate of oxidatively damaged mitochondria, a significant proportion was irreversibly removed by lysosomal activation, an event that has been previously described [49]. Cr supplementation prevented these severe ROS-dependent injuries and alterations and preserved mitochondrial integrity, biogenesis, and function, and, notably, protection of mitochondria invariably coincided with the* restoration* of myoblast differentiation under oxidative stressing conditions.

In particular, confocal microscopy/image analyses showed that supplemental Cr preserved mitochondrial morphology, single unit integrity, and networking immediately after oxidative insult. Importantly, the prompt assembly of elongated mitochondrial networks is a necessary condition for mitochondrial biogenesis, and both events are critically involved in myoblast differentiation [48]. Hence, the Cr-mediated reduction of the mitochondrial network fragmentation reflects the efficacy of its protective potential.

The abovementioned protective effects promoted by Cr are likely to depend on multiple mechanisms, a notion in line with the well-established Cr pleiotropism [3, 12, 50–52]. For instance, the lower degree of H_2_O_2_-inflicted cardiolipin peroxidation, a marker of mitochondrial oxidative damage [53], is conceivable with the direct antioxidant capacity of Cr [15, 16], and indeed also Trolox reduces the extent of cardiolipin peroxidation. However, according to previous data [15], the antioxidant capacity alone is a condition which may favour cell survival but is insufficient to fully protect cells from myogenic arrest: such a conclusion can be drawn from the observation that Trolox-loaded H_2_O_2_-injured C2C12 cells are unable to complete the differentiation task. Thus, the effects promoted by Cr are more complex as compared to those of a mere* bona fide* antioxidant and can be explained on the basis of its established pleiotropism [3, 12, 50, 52].

Similarly, another central event is the Cr-related maintenance of the mitochondrial ΔΨ (Figure 2), that is, mitochondrial function, which in turn may depend on its antioxidant effect; the contribution to an increased cellular energy charge and utilization through the larger availability of CrP [15]; the stabilization of mitochondrial Cr kinase in its active octameric form, which is known to consolidate the structure of mitochondrial membranes and increase their resistance to ROS or hypoxia [3, 54]; and the protection of mitochondrial permeability transition pore complex [55].

The Cr-dependent preservation of mitochondrial integrity and function in oxidatively injured cells is further supported by the 2D mt-protein analysis. Mitochondria purified from oxidatively injured cells at DifD3 had a protein profile similar to that observed in mitochondria from undifferentiated myoblasts, pointing to the H_2_O_2_-related myogenic inhibition, while Cr supplementation resulted in a protein pattern highly similar to that of normally differentiating myoblasts.

In this study, we highlighted a further mechanism which may significantly contribute to the mitochondrially oriented Cr effects, namely, the activation of AMPK. AMPK is a pivotal energy sensor playing a central role in linking mitochondrial function to cellular metabolism [43, 56–59]. It is widely accepted that its pharmacological activation in resting muscle has deep effects on the expression of an array of genes related to mitochondrial biogenesis and function [60–62]. In our intoxication/protection paradigm, AMPK activation is complex since it is promoted by both H_2_O_2_ and Cr. In the case of H_2_O_2_, it activates AMPK* per se*, an event that might constitute a physiological response to ROS in an attempt to counteract the differentiation arrest through the induction of an adaptive pathway promoting, for example, mitochondrial biogenesis and oxidative metabolism, both functionally oriented to better energy recovery and homeostasis (see also below). However, despite this attempt, cells undergo complete differentiation arrest, suggesting that this cellular response is* per se* insufficient to counteract this stress.

According to previous reports by other groups [57, 58], here we report that Cr* per se* caused increased phosphorylation of AMPK (31% ± 2.7). More interestingly, this increase seems to be additive to that promoted by H_2_O_2_ (25% ± 4.71); indeed, in Cr-supplemented cells exposed to oxidative stress, AMPK phosphorylation increased by 66% ± 7.10, a condition that coincides with the recovery of a* quasi*-normal myogenic ability. Thus, it is conceivable that the sum of these two distinct AMPK activation instances, along with the other Cr benefits, is sufficient to significantly help cells to overcome the differentiative arrest imposed by H_2_O_2_. In other words, the increased and cumulative activation of AMPK may represent another relevant feature of Cr-pleiotropic nature, which concurs with the rescue of the differentiation capacity. In this regard, it is worth noting that although the role of AMPK phosphorylation in the myogenic process is not yet fully understood [12, 52], Fu et al. [59] described that transient AMPK activation by AICAR resulted in myogenin increase, promoting myogenesis in C2C12 cells. Incidentally, we too reported [15] an increase of myogenin upon Cr supplementation in the same differentiation setting as in the present study.

Moreover, here we also assessed the gene expression level of the “*master*” regulator of the mitochondrial biogenesis, namely,* PGC-1α* and* Tfam*, regulated by* PGC-1α*, required for mitochondrial biogenesis, which mirrors the changing levels of mtDNA in the cell and plays a crucial role in mtDNA maintenance [63, 64]. In particular, the expression level of these two transcription factors increased under all the conditions tested, as compared to controls, and may reflect a behaviour that is functionally convergent to that seen for AMPK. Hence, they may play a role in the promotion of mitochondrial biogenesis under oxidative stressing conditions and, in turn, in the* restoration* of the myogenic capacity. Mitochondrial DNA copy number reflects the extent of mitochondrial biogenesis, whose variations can be better appreciated at later differentiation times (i.e., DifD3). As expected, oxidative stress caused a significant reduction of mtDNA copies; Cr* per se* slightly increased mtDNA content according to its prodifferentiation capacity [12] and, more interestingly, prevented the drastic reduction of mtDNA caused by oxidative stress.

Data presented herein also indicate that Cr* per se* induces significant modifications of some parameters directly or indirectly associated with mitochondrial content and function, such as increased mtDNA copies, ΔΨ, AMPK phosphorylation,* PGC-1α* expression, and a slight augmentation of mitochondrial mass: these effects are likely to be involved in the well known anabolic action of Cr in skeletal muscle [12].

## 5. Conclusions

On the whole, our results highlight that AMPK activation and* PGC-1α* upregulation could be important for maintaining the myogenic wave and differentiation even in adverse stressing conditions such as oxidative insult. These effects could represent an important mechanism contributing to the benefits of supplemental Cr in those muscular diseases, where oxidative stress, mitochondrial impairment, and differentiative imbalance play a causative role, for example, muscular dystrophies, mitochondrial cytopathies, aging, disuse atrophy, and sarcopenia [6–8, 65].

In this study, we have identified a wide range of converging mitochondrial positive effects of Cr in C2C12 cells differentiating under normal or oxidative stressing conditions, which contribute to Cr pleiotropism at the basis of its myogenic and protective activities.

Indeed, these mitochondrial effects are likely to concur with the other cellular actions that we and others have identified as important in supporting the myogenic process and protecting its execution from oxidative insult, such as the mild direct antioxidant activity [16, 50], ameliorated cellular energy [15, 54], increased expression of muscle regulatory factors and of IGF-1 [15], decreased apoptotic susceptibility, and cell death [3].

Together, these conditions jointly favour protection and rapid mitochondrial recovery minimizing the effects of oxidative damage, thus restoring myogenesis.
